# Exploring the Interplay of Wavelength, Quantum Yield, and Penetration Depth in In Vivo Fluorescence Imaging

**DOI:** 10.1007/s10895-024-03985-2

**Published:** 2024-11-04

**Authors:** Meital Harel, Rinat Ankri

**Affiliations:** The Department of Physics, Faculty of Natural Science, Ariel University, Ariel, 40700 Israel

**Keywords:** Fluorescence imaging, Monte carlo simulations, Multiplexing, Optical properties of tissues, Penetration depth, Quantum efficiencies, Single photon avalanche diodes

## Abstract

The intricate interplay between the irradiation wavelength, the fluorophore quantum yield (QY) and penetration depth profoundly influences the efficacy of in vivo fluorescence imaging in various applications. Understanding the complex behavior of fluorescence in vivo, specifically how variations in wavelength affect the QY of commonly used dyes and the depth of imaging is crucial for optimizing fluorescence imaging techniques, as it directly impacts the accuracy and efficiency of imaging in biological tissues. In our study, we explore these dynamics through Monte Carlo simulations conducted under conditions reflective of wide-field fluorescence imaging, examining how variations in wavelength impact the dye’s QY and depth of imaging, and consequently, the fluorescence behavior. A transition in the exponential decay of the emission depth exponent is observed around the 500–600 nm range, indicating varying degrees of influence of depth on the fluorescence emission. The analysis of the fluorophore’s QY reveals wavelength-dependent variations, with the most significant impact observed in the 600–700 nm range. Moreover, we continued our investigation to explore multiplexing, unveiling insights into the spacing between identical spots in multiplexing images across various depths and wavelengths.

**Supplementary Information:**

The online version contains supplementary material available at 10.1007/s10895-024-03985-2.

## Introduction

Imaging laboratory animals through fluorescence is a useful technique widely employed in biomedical research for visualizing cellular and molecular processes in vivo [1]. Typically conducted within the wavelength range of 200 to 750 nm, fluorescence imaging offers high sensitivity and specificity, enabling researchers to track labeled molecules or cells with exceptional precision [2]. However, the depth of penetration achievable within this spectral range is limited, generally confined to superficial layers of tissue, typically up to a few millimeters. Consequently, deep-seated structures may be challenging to visualize accurately. Imaging at these wavelengths often encounters issues such as autofluorescence from endogenous molecules and scattering of light by surrounding tissues, leading to reduced image contrast and resolution [3]. Studies have shown that NIR fluorophores enable non-invasive imaging at depths of several centimeters within tissue, making them highly effective for surgical imaging, tumor detection, and the monitoring of biological processes [4, 5].

Understand the wavelength-dependence optical characterization of fluorophores is of high importance in order to improve fluorescence-based imaging. It allows for fine-tuning their spectral properties, to enable precise targeting and multiplexing in imaging applications. Discerning the quantum yield provides important insights into the efficiency of fluorescence emission, facilitating the development of brighter and more sensitive probes. In particular, unraveling the relationship between penetration depth and these parameters is pivotal for optimizing imaging depth in biological tissues and enhancing signal-to-noise ratios, as well as to help researchers to craft fluorophores with improved optical characteristics. Among prevalent fluorophore families like Cyanins, Atto, or Alexa, each exhibits distinct quantum efficiency profiles. For instance, Cyanin dyes [6] often boast high quantum efficiencies, reaching values of 0.80 or higher, particularly in the visible spectrum. Atto dyes [7], known for their exceptional brightness and stability, typically exhibit quantum efficiencies ranging from 0.50 to 0.80 across a wide range of wavelengths. In comparison, fluorophores from the Alexa family [7], prized for their brightness and photostability, often display quantum efficiencies around 0.60 to 0.70, with optimized performance in the near-infrared spectrum. These varying quantum efficiency values underscore the importance of selecting the appropriate fluorophore for specific imaging applications, considering both wavelength requirements and desired imaging performance.

In our study, we aim to present the expected fluorescence behavior of fluorophores in the visible-near infrared (NIR) regimes that vary in their optical properties, such as wavelength (WL), quantum yield (QY), and fluorescence lifetime (FLT). NIR light penetrates several millimeters into human skin, making it ideal for non invasive imaging deeper tissue layers. Through Monte Carlo (MC) simulations and their analysis, we anticipate elucidating how variations in wavelength influence both quantum yield and penetration depth, thereby providing invaluable insights into the design and optimization of fluorophores for diverse applications. We determine the fluorophore properties that allow for the highest penetration depth in in vivo fluorescence-based imaging. Following this, we investigate the multiplexing imaging performance of the two dyes that presented the optimal optical properties for tissue depth. This investigation holds promise for not only expanding our theoretical understanding of fluorescence, but also for driving practical advancements in fields such as biomedical imaging, materials science, and environmental sensing.

## Methods – Monte Carlo Simulations

Employing a MC methodology, outlined in our recent publication [8], we adhere closely to a robust protocol governing photon propagation within biological tissues [9]. This approach is underpinned by two core assumptions, briefly elucidated herein: (i) exciting photons were neglected and (ii) temporal emission decay was recorded. Within the framework of a semi-infinite tissue structure (unlimited in dimensions x and y), the precise placement of the fluorophore at a designated depth, z, is meticulously executed. Photon emission simulation follows an exponential decay pattern, denoted by the equation:

1 $$\:N\left(t\right)=A\cdot\:{exp\:}^{-\frac{t}{LT}}$$

where A represents the pre-exponential factor, derived from the initial photon count (a) multiplied by the QY of the fluorophore, and LT signifies the fluorophore’s lifetime. Each emitted photon propagated in the tissue, according to its scattering and absorption properties, for a nonspecific period of time, then was either absorbed or exited the tissue surface into the air $\:(n=1)$. Photons reaching the detector were counted, within a given pixel, while the total counted photons were multiplied by the SPAD array photon efficiency (PE, determined as 13%, corresponding to the SPAD array PE used in our experiments, see below). Once all photons emitted from a given time point on the decay curve have completed their propagation, the next emission occurs. This iterative simulation yields 117 time-sampled intensity images for each scenario, culminating in the synthesis of distinct intensity images for various experimental conditions. The 117 images were combined to create the intensity image of the fluorescence.

In our simulation study, conducted under conditions reflective of a single photon avalanche diodes (SPAD) camera, we explored a range of depths spanning from 0.2 to 1.4 cm, incrementally increasing by 0.2 mm intervals. Across these depths, we examined the fluorescence emission intensity at four distinct wavelengths: 500 nm, 600 nm, 700 nm, and 800 nm. These wavelengths were chosen to represent specific regions within the visible and near-infrared spectrum, each bearing significance in various biomedical and optical applications. We conducted simulations on selected QY values, aiming to provide a comprehensive understanding of the relationship between wavelength, fluorophore efficiency and depth. The parameter values used in our simulations are listed in Table 1. The parameters and their sources are as follows: RI – refractive index [10], µ_a_ – absorption coefficient [10], µ_s_‘– reduced scattering coefficient [10], g - the anisotropy factor, due to the scattering events of the photons in the tissue [11], QY – fluorophore quantum yield [12] (we chose to use the QY of representative Alexa Fluor dyes, very commonly used in biomedical and biological research) and PE - Photon Efficiency [13], while the PE is for the simulated SPAD array. The column representing QY denotes the maximum value we used, chosen according to the ATTO fluorophores [12]. Our simulations encompassed different values up to this documented maximum, ensuring comprehensive coverage across the spectrum of potential QY outcomes. Details of the simulated QY values can be found in Supporting Information, Table S1.

**Table 1:** Optical property values for human skin epidermal tissue used in our simulations. The PE is for the SPAD array

λ (nm)	RI	µ_a_ (cm^− 1^)	µ_s_’ (cm^− 1^)	g	QY	PE (%)
500	1.44	1.19	32.5	0.76	0.92	50
600	1.43	0.69	21.8	0.79	0.66	40
700	1.42	0.48	16.7	0.82	0.36	27
800	1.41	0.43	14	0.84	0.12	13

## Results and Discussion

**Fig. 1:**
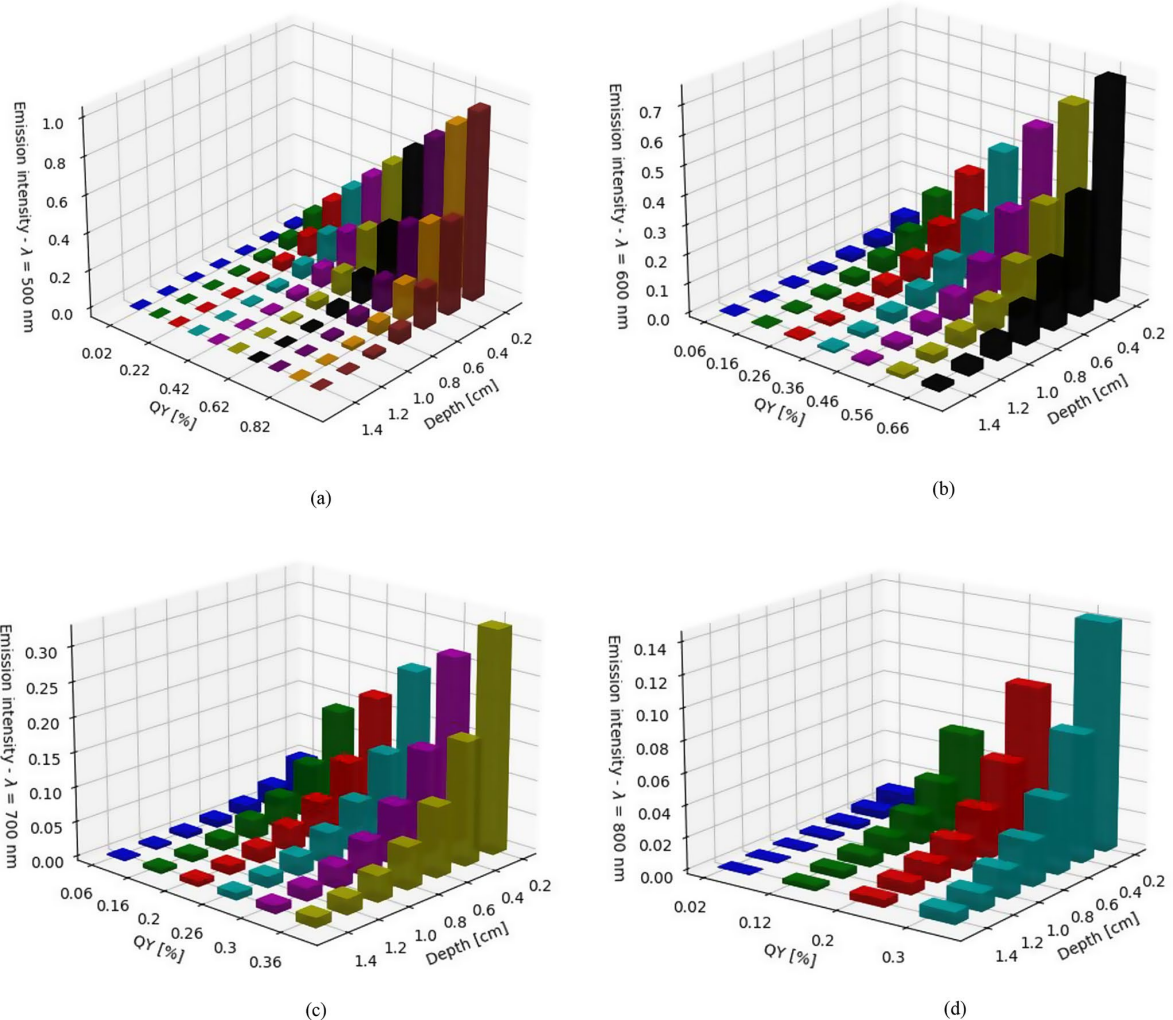
Normalized histograms of detected emission based on the number of illuminated pixels. The distributions are presented across depth and QY for various wavelengths: (**a**) 500 nm, (**b**) 600 nm, (**c**) 700 nm, and (**d**) 800 nm

We initially used our simulations to examine the impact of the fluorophore’s QY on the fluorescence intensity across different wavelengths in the visible to near-infrared ranges and at various depths. We constructed normalized histograms to depict the distribution of detected emission concerning both depth and QY, generating individual histograms for each wavelength (Fig. 1). The emission intensities were normalized according to the highest intensity achieved for $\:\lambda\:=500\:nm.$

Figure 1 demonstrates that the lowest wavelength yields the highest emission intensity, while the highest wavelength results in the lowest emission intensity. This is also due to the fact that the highest quantum efficiency of the fluorophore corresponds to the longest wavelength, resulting in a higher number of detected emissions. This discrepancy underscores the relationship between fluorophore properties and excitation conditions, which directly impact imaging outcomes. Additionally, shallower depths exhibit more detected emission compared to deeper depths. These findings are expected and validate our simulation for the different optical properties of the tissue in the different wavelengths.

**Fig. 2:**
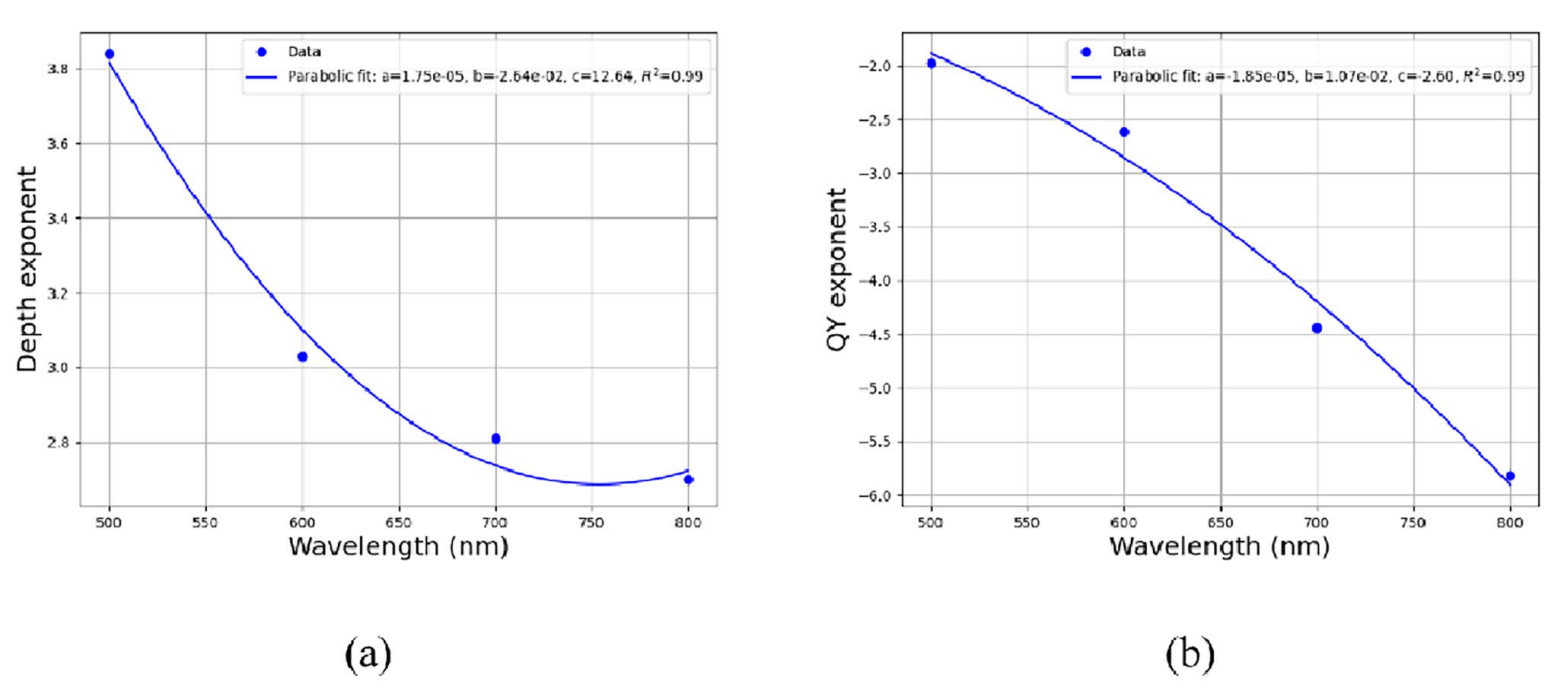
Depth and QY exponents analysis: (**a**) Depth exponent trends across different wavelengths. (**b**) QY exponent variations with respect to wavelength

Based on these simulation’s results, we then study the influence of the fluorophore’s QY and depth of imaging on the number of detected fluorescence photons. To do so, an exponential decay was plotted for each histogram in Fig. 1, while the exponential decay of the emission vs. depth was named as “Depth exponent” and the decay for the QY vs. wavelength was names ad “QY exponent”. The resulted curves are presented in Figure S1 of the *Supporting Information*. Analyzing the depth exponent, represented by the parameter ‘b’ in the decay equation $\:y=a\cdot\:\text{e}\text{x}\text{p}(-b\cdot\:x)$ of each curve in Fig. S1(a-d), sheds light on the relationship between normalized detected emission and depth, across different wavelengths. Derived from fits presented in Figure S1(a-d), the results for the “b” component the “Depth exponent”, were collected and averaged for each wavelength (see Fig. S2(a) in the Supplemantary information). Then, a polynomial fit was calculated and presented in Fig. 2(a).

These findings reveal a distinct decrease in the depth exponent from 500 to 600 nm, indicating a diminishing influence of depth on pixel illumination within this wavelength range. Starting at 600 nm, a more moderate decrease emerges, implying reduced sensitivity to depth changes at longer wavelengths. These observations underscore a wavelength-dependent variation in the interplay between depth and detected emission, with a discernible transition around the 500–600 nm range, subsequently leading to a relatively moderate trend at longer wavelengths.

The same analysis was executed for the dependence in the QY values. We analysis the “b” parameter in the equation $\:y=a\cdot\:\text{e}\text{x}\text{p}(-b\cdot\:x)$, where “y” represents the normalized emission intensity and “x” represents the QY. These results are extracted from fits presented in Figure S1(e-h) in the *Supplemantary information*, where the normalized values were plotted against QY for different wavelengths. These “b” results, which we named as “QY exponent”, were collected and averaged for each depth (see Fig. S2(b) in the *Supplemantary information*). Then, a polynomial fit was calculated and presented in Fig. 2(b).

The graph illustrates a decrease in the ”QY exponent”, indicative of the influence of QY on detected emission, varies across different wavelength ranges. Notably, between 500 and 600 nm, the decrease in values is relatively small, suggesting a moderate effect of QY on detected emission within this range. However, between 600 and 700 nm, the decrease in ”QY exponent” is most pronounced, indicating a significant impact of QY on detected emission within this wavelength interval. Finally, between 700 and 800 nm, the decrease in the values is moderate, suggesting a somewhat reduced influence of QY on detected emission compared to the 600 to 700 nm range. These observations highlight the wavelength-dependent variability in the relationship between QY and detected emission, with the most substantial effect observed in the 600 to 700 nm range, followed by relatively smaller effects at other wavelengths.

The parameter “a”, which is the coefficient of the” x^2^” term in our parabolic fit model, was found to be 1.75 for the depth exponent as a function of wavelength (see Fig. 2(a)). This value is smaller than the corresponding “a” parameter of 1.85 for the QY exponent versus wavelength (see Fig. 2(b)). This indicates that the dependence of QY on wavelength is stronger than that of the depth exponent, as evidenced by the more pronounced quadratic relationship in the QY data. The high R^2^ value of 0.99 for both fits indicates an excellent accuracy. Therefore, the variation in quantum yield with changes in wavelength is more significant, suggesting a greater sensitivity of QY to wavelength compared to the depth exponent.

**Fig. 3:**
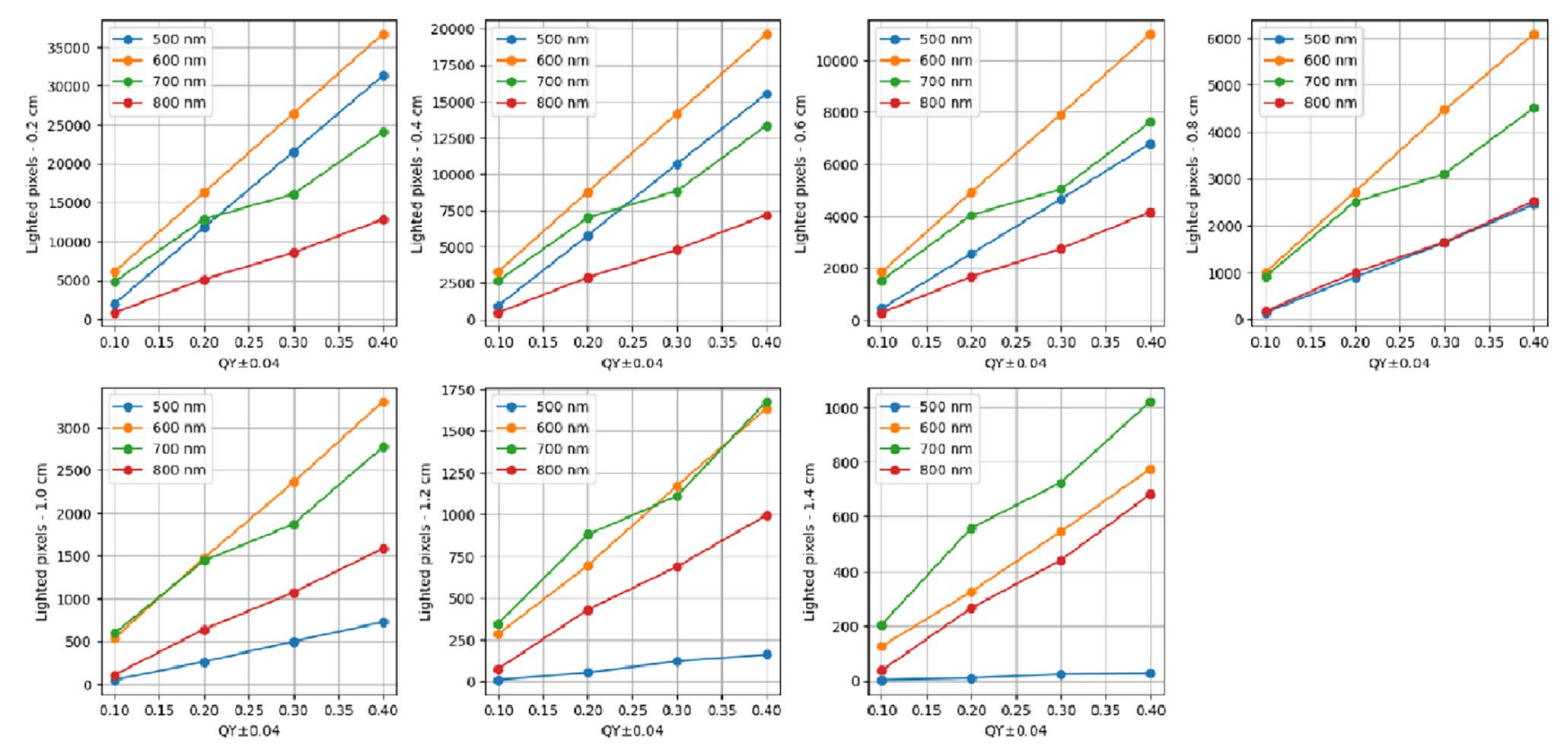
Relationship between detected emission and QY for various wavelengths across depths ranging from (**a**) 0.2 cm to (**g**) 1.4 cm

**Fig. 4:**
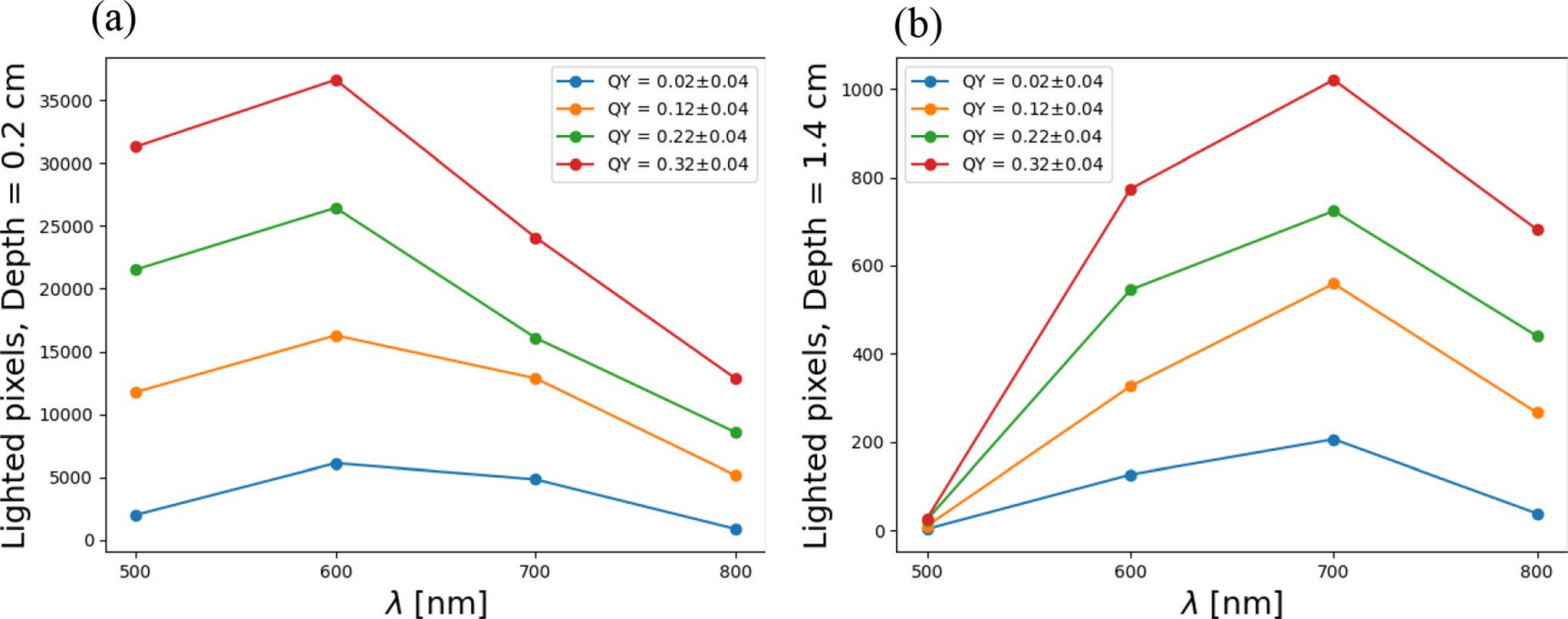
Relationship between detected emission and wavelengths for various QY across depths (**a**) 0.2 cm and (**b**) 1.4 cm

However, upon closer examination, an intriguing behavior emerges from Fig. 1, as presented in Figs. 3, and 4 (as well as Videos S1 and S2 in the *Supporting Information*). These figures and videos consistently compare QY values across different scenarios. Given that identical QY values across all wavelengths are uncommon, we used a range of values (± 0.04) on the x-axis, as shown in Fig. 3. This figure illustrates the relationship between detected emission and QY for various wavelengths at depths ranging from (a) 0.2 cm to (g) 1.4 cm.

Two noteworthy phenomena emerge from the data presented at Fig. 3. First, it is surprising to find that even near the surface, at a depth of 0.2 cm, the count of detected emission for a wavelength of 500 nm is lower than that of 600 nm, since the scattering and absorption coefficients at 600 nm are significantely lower than at 500 nm. For lower QY values, it is even lower than the 700 nm wavelength. As shown in Table 1, several variables contribute to the observed outcomes, each undergoing changes that complicate pinpointing the exact factors responsible for the situation of low results for 500 nm. Second, an intriguing shift occurs with the 700 nm wavelength: surpassing the count of detected emission associated with the 600 nm wavelength at approximately 0.8 cm depth. At 1 cm and beyond, the count for the 700 nm wavelength eclipses that of the 600 nm wavelength, indicating a higher efficacy of the former in deeper scenarios. Figure S3(a-g) and Video S2 (in the *Supporting Information* section) showcase the outcomes pertaining to the correlation between the quantity of detected emission and QY across various wavelengths.

As a complement to Figs. 3 and 4 presents the relationship between detected emission and wavelengths for various QY values and shows representative plots for these relationships at depths of (a) 0.2 cm and (b) 1.4 cm. The results for the intermediate depths are provided in Fig. S3 in the Supporting Information section. As explained, the values for QY represent values ± 0.04.

Figure 4 along with Figure S3 and Video S2, in the *Supporting Information*, demonstrate that the number of detected emission increases with the increase in the fluorophore’s QY, across all depths. This result was expected. Yet, an interesting behavior was revealed when considering depths beyond 1 cm. Up to a depth of 1 cm, the 600 nm wavelength yields the maximum value. However, beyond this depth, a crossover occurs, with the 700 nm wavelength attaining the maximal value for depths exceeding 1.4 cm.

A possible explanation can be found in our recent paper, in which we demonstrated that light scattering within tissue interfered with the penetration of infrared light [8]. As we probed deeper, we expected increased scattering to hinder photon movement. However, these graphs reveal that beyond a certain critical point, scattering intensity diminishes (consistent with previous studies [8]), resulting in insufficient photons emerging from the tissue. Meaning, we found that scattering actually facilitates the light beam’s emergence from the tissue.

**Fig. 5:**
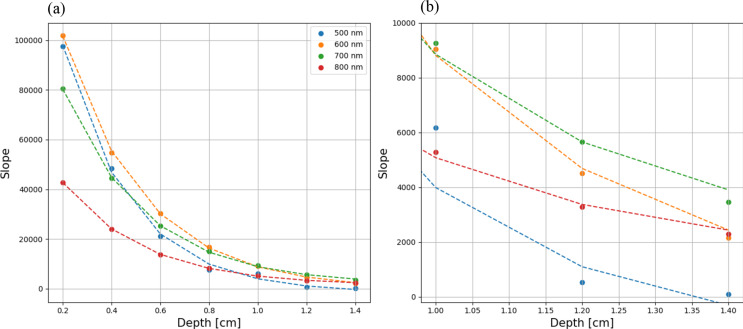
The slope of variation of detected emission and QY across depths and wavelengths: (**a**) illustrates slope variations (as defined in the previous paragraph) with depth for multiple wavelengths. The exponent fits are − 3.59 for 500 nm, -3.02 for 600 nm, -3.01 for 700 nm, and − 2.99 for 800 nm; (**b**) zooms in on the deepest depths only

To determine the dependency of detected emission on QY, we calculated the slope of each plot in Fig. 3. This slope provides valuable insight into the relationship between fluorescence intensity and QY. Subsequently, we plotted these slopes against depth for various wavelengths, offering a comprehensive analysis of how depth influences the correlation between detected emission and QY across different spectral regions. Results are presented in Fig. 5.

Figure 5 reveals that depth, governed by scattering processes, obscures the influence of both wavelength and QY on the number of detected emission. At shallower depths, the dependency on QY is notably pronounced within the 500–600 nm range, followed by 700 nm, with a substantial gap leading to 800 nm, where the impact of QY on detected emission diminishes. However, around the 0.8 cm mark, the significance of depth on detected emission appears to wane, aligning with our findings [8]. These observations underscore the complex interplay between depth, wavelength, and QY in fluorescence imaging, offering crucial insights for refining imaging techniques and informing future research directions in this domain. Figure 5 uncovers some interesting observations: First, the slopes of the graphs indicate that the dependence varies significantly near the surface, but as depth increases, the change in dependence diminishes. This implies that deep inside the tissue, choosing the wavelength is less crucial. Another piece of evidence for this is the substantial variance near the surface (100,000 for 600 nm compared to 40,000 for 800 nm), contrasted with much closer results deep inside the tissue (4,000 for 700 nm compared to 0 for 500 nm). Second, surprisingly, at depths close to the surface, the strongest dependence is observed at 600 nm, followed by 500 nm. Third, deeper within the tissue, the strongest dependence is observed at 700 nm, while 600 nm and 800 nm follow closely and yield similar results.

**Fig. 6:**
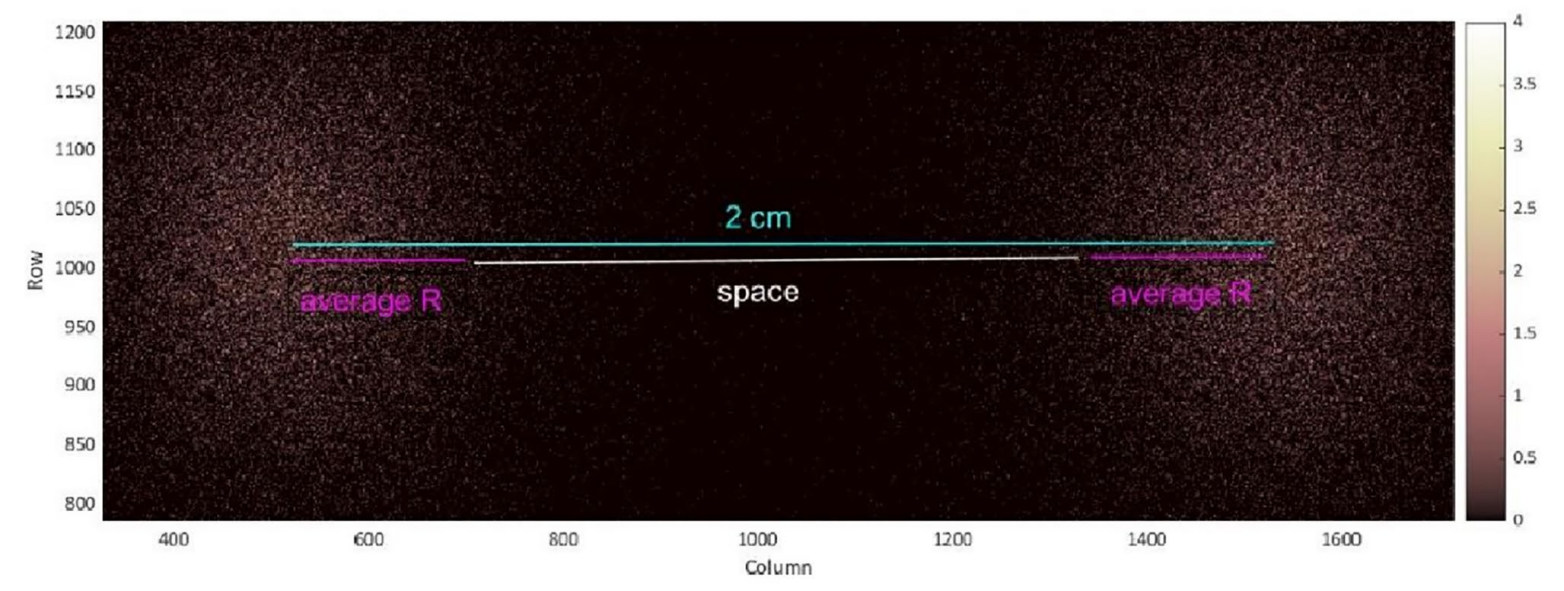
Two identical spots in a multiplexing image at a depth of 0.4 cm with a wavelength of 600 nm and QY = 0.66. The fluorophores were deposited with a vertical distance of 2 cm (light blue markers), and the average radius of each spot is shown in pink. The white line indicates the resulting space between the spots

An interesting metric to consider is the spacing between two identical spots in a multiplexing image, measured at various depths and wavelengths, for multiplexing purposes. Figure 6 is a representing image of two identical spots positioned at a depth of 0.4 cm, with a wavelength of 600 nm and a QY of 0.66. These values were chosen because they are representative. The fluorophores were deposited with a vertical distance of 2 cm (indicated in light blue), and the average radius [8] of each spot is highlighted in pink. The resulting white line represents the spacing between the two spots.

**Fig. 7:**
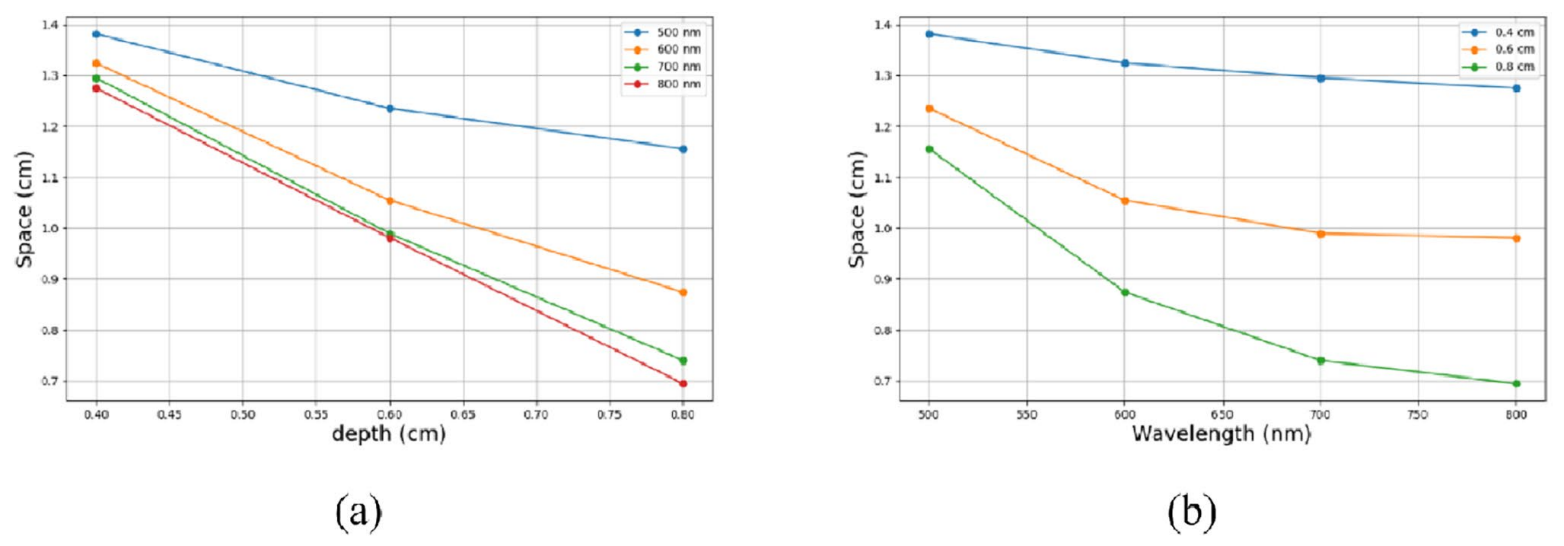
The space between two identical spots for various depths and wavelengths. (**a**) The space vs. Depth for various wavelengths. (**b**) The space vs. Wavelength for various depths

Multiplexing simulations were performed for additional wavelengths [6], and the space between the spots at various depths and for different wavelengths were extracted. The results for the space in various cases are shown in Fig. 7.

Figure 7 reveals that the spacing decreases with both increasing depth and wavelength, with the influence of wavelength diminishing at higher wavelengths. Meaning that as depth and wavelength increase, the dispersions also increase, leading to a larger average radius of each spot and consequently reducing the spacing between them.

## Conclusions

The complex interaction between irradiation wavelength, QY, and penetration depth significantly affects the effectiveness of in vivo fluorescence imaging across various applications. In our study, we investigated these dynamics using Monte Carlo simulations that replicate in vivo fluorescence imaging conditions, analyzing how changes in wavelength influence the dye’s QY and imaging depth, and subsequently, the fluorescence behavior.

We validated our simulation (Fig. 1) by achieving the expected values for detected emission across different optical properties of the tissue, such as depth and QY, for various wavelengths. Then (Fig. 2(a)), we defined the “Depth exponent,” which revealed a distinct decrease from 500 to 600 nm, indicating a diminishing influence of depth on pixel illumination within this wavelength range. We also defined the “QY exponent” (Fig. 2(b)) and found that the influence of QY on detected emission varies across different wavelength ranges: there is a moderate effect between 500 and 600 nm, a significant impact between 600 and 700 nm, and a somewhat reduced influence between 700 and 800 nm. We compared the coefficients of the Depth polynomial fit with those of the QY polynomial fit and found that the dependence of QY on wavelength is stronger than that of the Depth exponent. In Fig. 3, at a depth of 0.2 cm, the detected emission count for a wavelength of 500 nm was found to be lower than that of 600 nm. Additionally, in deeper scenarios, the 700 nm wavelength exhibited higher efficacy. Figure 4 sharpened the insight so that up to a depth of 1 cm, the 600 nm wavelength produces the highest emission count. However, beyond this depth, a crossover occurs, with the 700 nm wavelength achieving the highest values for depths greater than 1.4 cm. The outcome was that scattering actually facilitates the light beam’s emergence from the tissue. Figure 5 reveals that the dependence between depth, wavelength, and QY varies significantly near the surface, but as depth increases, the change in dependence diminishes. This implies that deep inside the tissue, choosing the wavelength is less crucial. Finaly, we examined the spacing between two identical spots in a multiplexing image, and found that as depth and wavelength increase, the dispersions also increase, leading to a larger average radius of each spot and consequently reducing the spacing between them.

The detailed investigation of the interplay between irradiation wavelength, quantum yield (QY), and penetration depth in in vivo fluorescence imaging introduces several novel concepts, such as the “Depth exponent” and the “QY exponent,” which elucidate how depth and QY impact fluorescence emission across different wavelength ranges. We demonstrated that the influence of wavelength on detected emission varies, with distinct patterns observed for wavelengths between 500 and 800 nm. Our findings highlight that, at shallow depths, wavelength selection significantly affects fluorescence emission, whereas at greater depths, this effect diminishes. This research also uncovers that scattering can enhance light emission from tissues and reveals how wavelength and depth impact the spatial dispersion of fluorescence in multiplexed imaging. These insights pave the way for optimizing fluorescence imaging techniques and improving the accuracy of diagnostic and research applications involving tissue imaging.

## Electronic Supplementary Material

Below is the link to the electronic supplementary material.

Supplementary Material 1

## Data Availability

Research data can be shared by the authors upon request.
