# Supplementary material for: Exploring the Interplay of Wavelength, Quantum Yield, and Penetration Depth in In Vivo Fluorescence Imaging
Source: J Fluoresc. 2024 Nov 4;35(8):6553–61. doi: 10.1007/s10895-024-03985-2 (PMC12476312; doi:10.1007/s10895-024-03985-2)
Supplement: Supplementary file 1 — Supplementary Material 1 [file 10895_2024_3985_MOESM1_ESM.docx]

**Supporting Information**

Exploring the Interplay of Wavelength, Quantum Yield, and Penetration Depth in *In Vivo* Fluorescence Imaging

Meital Harel, Rinat Ankri*

–––––––––

Faculty of Natural Science, Ariel University, Ariel 40700, Israel

*Correspondence

Email: rinatsel@ariel.ac.il

**Content**

Table S1: Summary of simulation QY parameters.

Figure S1: Depth and Quantum Yield Exponents Analysis: (a-d) Analysis of normalized detected emission with respect to depth across various Quantum Yield (QY) values and exponential decays, depicted for different wavelengths: (a) λ=500 nm, (b) λ=600 nm, (c) λ=700 nm, and (d) λ=800 nm. (e-h) Examination of normalized detected emission concerning QY for different depths and exponential decays, illustrated for various wavelengths: (e) λ=500 nm, (f) λ=600 nm, (g) λ=700 nm, and (h) λ=800 nm.

Figure S2: The detailed calculations derived from the fits presented in Figure S2. (a) Normalized results vs. depth. (b) Normalized results vs. QY.

Video S1: Relationship between detected emission and QY for various wavelengths across depths ranging from (a) 0.2 cm to (g) 1.4 cm.

Figure S3: Relationship between detected emission and wavelengths for various QY across depths ranging from (a) 0.2 cm to (g) 1.4 cm.

Video S2: Relationship between detected emission and wavelengths for various QY across depths ranging from (a) 0.2 cm to (g) 1.4 cm.

Table S1 summarizes the simulation QY parameters.

| **WL (nm)** | **QY** | **QY-0.10** | **QY-0.20** | **QY-0.3** | **QY-0.4** | **QY-0.5** | **QY-0.6** | **QY-0.7** | **QY-0.8** | **QY-0.9** |
| --- | --- | --- | --- | --- | --- | --- | --- | --- | --- | --- |
| 500 | 0.92 | 0.82 | 0.72 | 0.62 | 0.52 | 0.42 | 0.32 | 0.22 | 0.12 | 0.02 |
| 600 | 0.66 | 0.56 | 0.46 | 0.36 | 0.26 | 0.16 | 0.06 |  |  |  |
| 700 | 0.36 | 0.26 | 0.16 | 0.06 |  |  |  |  |  |  |
| 800 | 0.12 | 0.02 |  |  |  |  |  |  |  |  |

**Table S1.** Summary of simulation QY parameters.

Figure S1 presents the decay of the normalized emission intensity versus depth, for various QY values (a-d). Panels (e-h) in Figure S1 show the exponential behavior for different depths. Panels (e-h) in Figure 1 show the exponential fits for different depths.

| 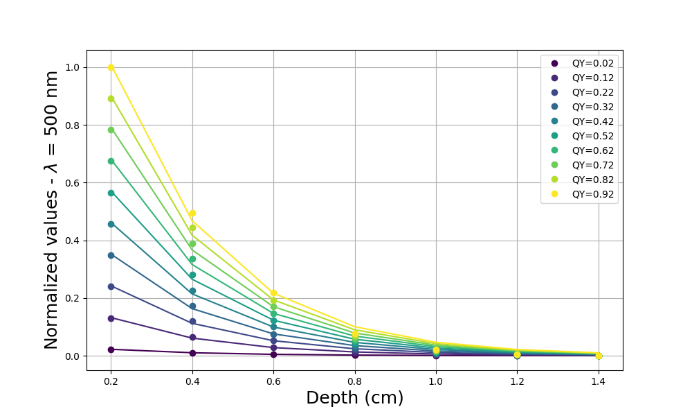 | 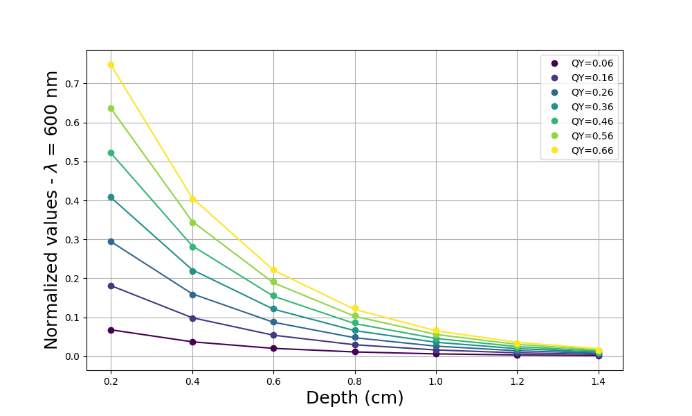 |
| --- | --- |
| (a) | (b) |
| 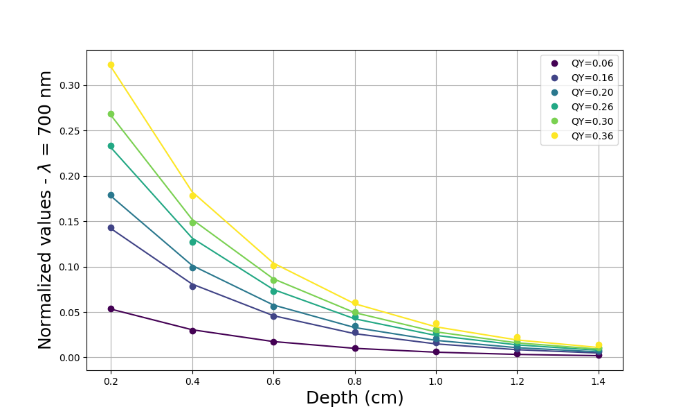 | 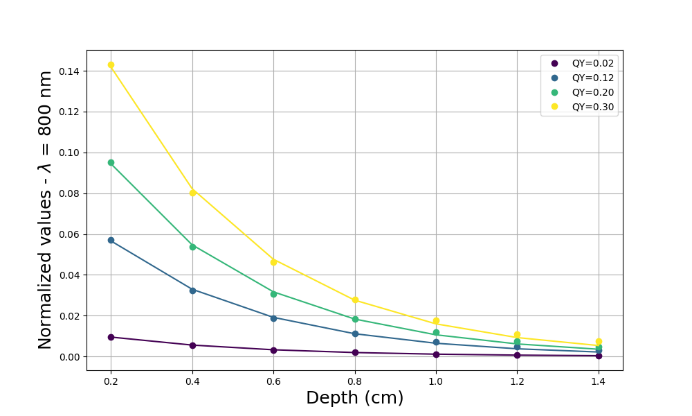 |
| (c) | (d) |
| 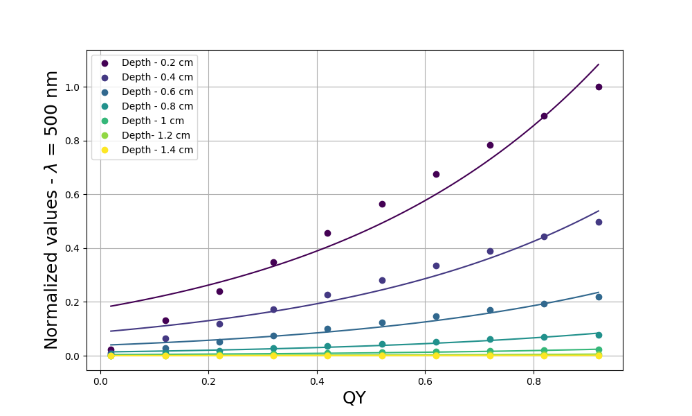 | 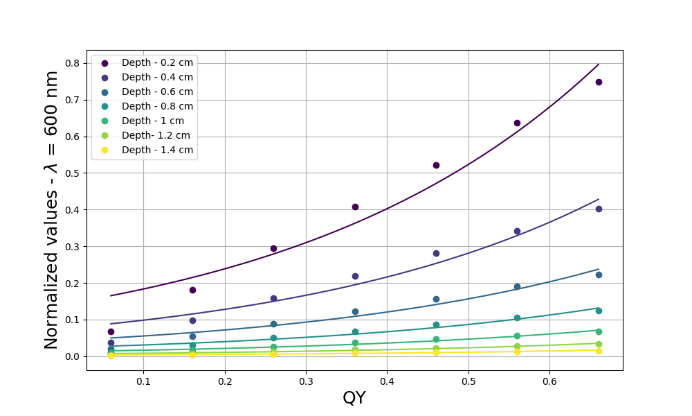 |
| (e) | (f) |
| 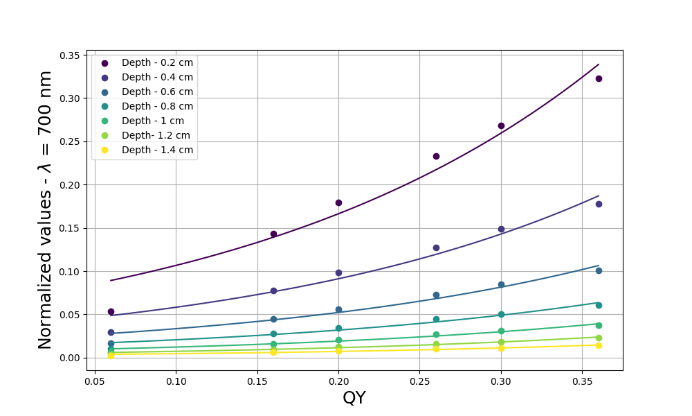 | 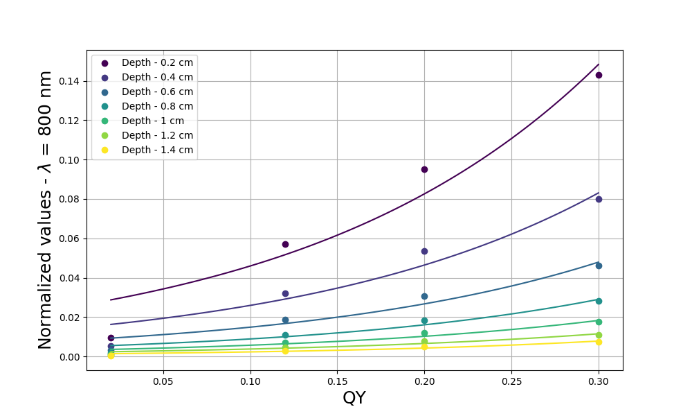 |
| (g) | (h) |

**Figure S1**. Depth and Quantum Yield Exponents Analysis: (a-d) Analysis of normalized detected emission with respect to depth across various Quantum Yield (QY) values and exponential decays, depicted for different wavelengths: (a) λ=500 nm, (b) λ=600 nm, (c) λ=700 nm, and (d) λ=800 nm. (e-h) Examination of normalized detected emission concerning QY for different depths and exponential decays, illustrated for various wavelengths: (e) λ=500 nm, (f) λ=600 nm, (g) λ=700 nm, and (h) λ=800 nm.

The detailed calculations derived from the fits presented in Figure S1 are shown in Figure S2. The results for the “b” in the equation $y=a\cdot\exp\left( -b\cdot x \right)$ were collected and averaged for each wavelength. Then, a polynomial fit was calculated and presented in Fig. 2 in the main article.

(a)

(b)

**Fig. S2.** The detailed calculations derived from the fits presented in Figure S2. (a) Normalized results vs. depth. (b) Normalized results vs. QY.

The Relationship between detected emission and QY for various wavelengths across depths ranging from (a) 0.2 cm to (g) 1.4 cm is presented as a movie in Video S1.

[..\Lights vs QY.mp4](file:///C:\Users\rinatankri\Lights%20vs%20QY.mp4)

**Video S1.** Relationship between detected emission and QY for various wavelengths across depths ranging from (a) 0.2 cm to (g) 1.4 cm.

| 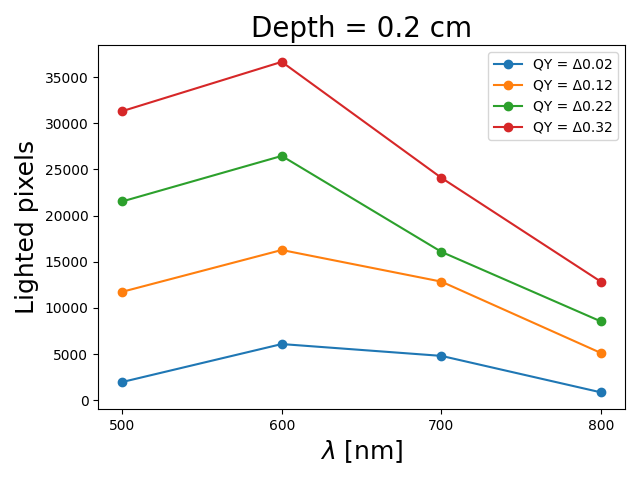 | 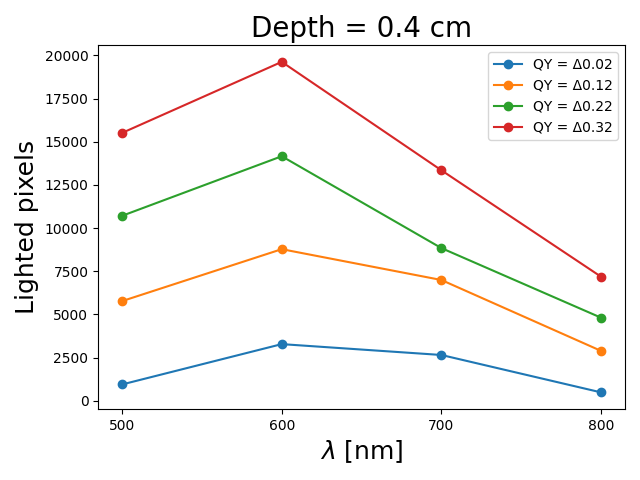 |
| --- | --- |
| (a) | (b) |
| 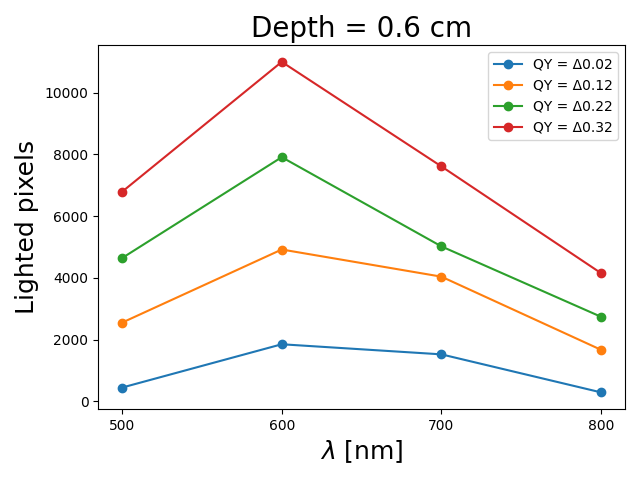 | 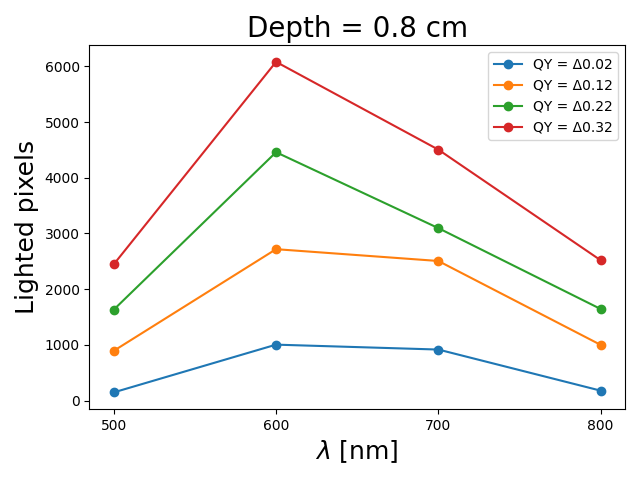 |
| (c) | (d) |
| 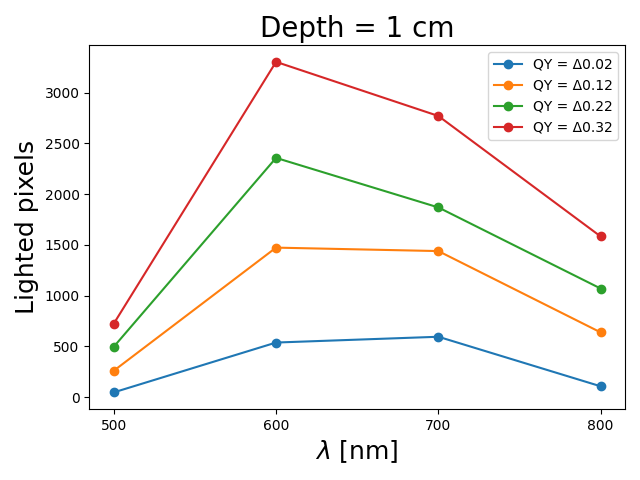 | 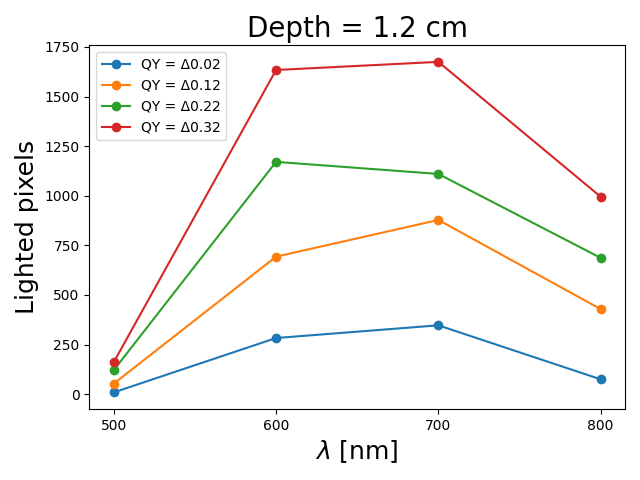 |
| (e) | (f) |
| 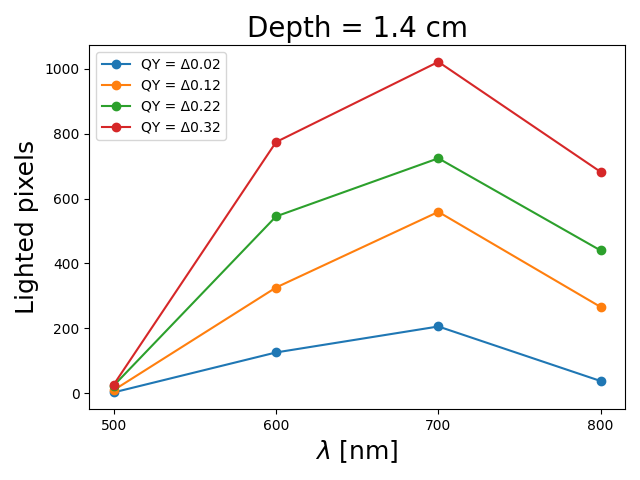 |  |
| (g) |  |


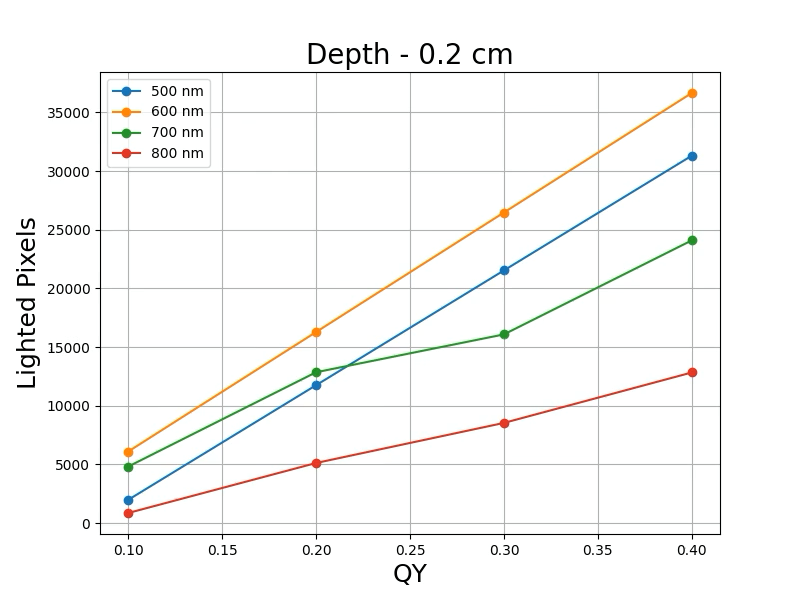


**Figure S3.** Relationship between detected emission and wavelengths for various QY across depths ranging from (a) 0.2 cm to (g) 1.4 cm.

The Relationship between detected emission and wavelengths for various QY across depths ranging from (a) 0.2 cm to (g) 1.4 cm is presented as a movie in Video S2.

[..\Lights vs WL.mp4](file:///C:\Users\meita\Documents\optical%20properties\Lights%20vs%20WL.mp4)

**Video S2.** Relationship between detected emission and wavelengths for various QY across depths ranging from (a) 0.2 cm to (g) 1.4 cm.
